# On the Good Effects of Resuscitated Salivation

**Published:** 1813-07

**Authors:** G. D. Yeats

**Affiliations:** Bedford


*To the Editors of the Medical and Physical Journal.*

GENTLEMEN,

GENTLEMEN,

I INTRODUCE the following case to you for insertion in your Journal, if you think it worthy such insertion. It appears to me especially instructive in one point of view, although a perfect restoration to health has not taken place, that of the advantages to be derived from the re-introduction of of mercury into the system when the most distressing symptoms had occurred during the original use of that mineral, and had continued in the most painful way for about fourteen months. Isolated cases do not in general contribute to our experience, except they come in confirmation of the observations of others. For this, among other reasons, the case deserves attention, as strengthening the remarks and practical information of Sylvester*, Dobson†, and Bardsley‡, on resuscitated Salivation.

I am, Gentlemen,

*Bedford*, Your very obedient Servant,

*June* 1, 1813. G. D. YEATS, M. D.

*On the good Effects of resuscitated Salivation.*

March 2, 1812.—E. P. æt. 22, a female, complains of a tremulous sensation at the pit of the stomach about once or twice in twenty-four hours, attended by a fainting disposition, and cold perspiration in considerable quantity. Sometimes passes a day without such sensations. On these attacks she takes a little cold water, which, though immediately rejected, relieves the uneasy feel at the stomach. Is frequently troubled with a giddiness, heaviness, and pain in the head—complains also that nothing stays on her stomach; whatever is taken of any description causes a weight and tightness at the stomach, until thrown up again; and the rejected matter is always in a very acid state, and in much greater quantity than what is taken. Is never sick except something is taken into the stomach. Has no appetite but when she sees food that she fancies, such as bread-pudding, nauseating the idea of meat. She feels a disposition to eat, but in indulging the disposition, the above sensations of weight and tightness, with consequent vomiting, are instantly produced. When the ingesta are first thrown up, it is with scarcely any effort, but afterwards a good deal of struggle takes place with the retching, and much clear liquid, extremely acid, is vomited, with a little phlegm. Has very considerable thirst, with a sensation of heat at the scrobiculus cordis, which is allayed by half a pint of cold water, although it is immediately rejected. Once she took a little warm rum and water, which caused instantly great pain with a violent burning heat, aggravating all the uneasy sensations at the stomach, followed by profuse perspiration and vomiting as usual. Her agony was such that she appeared to her friends to be dying. Bowels costive; urine

* Med. Observ. and Inquiries, vol. iii. pp. 241-56.
† Ibid. pp. 174-9. ‡ Med. Reports.

highhigh-colored. She states her stools to be dark-colored. Pulse 76, feeble.

E. P. has been in this state of symptoms for about fourteen months, and they commenced under the following circumstances. She complained at that time (December 1810) of a great tightness all round the waist, with a swelling on the region of the stomach towards evening, to such an extent as to oblige her to unlace her clothes; dyspnœa; occasional pain about the stomach; appetite indifferent; no thirst; no vomiting, nor sickness of stomach; and no particular uneasiness after taking food. Previous to the accession of these symptoms, she had been gradually failing: her ankles had swelled about the preceding May, and she had felt languid. She says these complaints were brought on by living on a watery vegetable diet, to get rid of some pimples in her face, but, notwithstanding, she grew lusty. The swelled ankles and languid feel went off on a return to a more generous diet, and she remained well till about the period when the train of symptoms just described commenced. She began to be regular in her nineteenth year, and had always enjoyed perfect health, never having been ill except having had the small-pox in the fourth year of her age. She continued perfectly regular in her menstrual discharge till September 1810, when it ceased, and returned in the following January, and appeared twice till May, since which time it has never made its appearance.

In September, 1810, a physician was consulted. He ordered various remedies, which the nature of the symptoms indicated; but these not succeeding, she was put upon a course of mercury in the beginning of December following, both by friction and pills, which, in about three weeks, brought on a very considerable salivation, which lasted three weeks. On the commencement of the salivation, she felt a constant inclination to make water, with a bearing down pain and straining at stool, which continued in a greater or less degree for a month, but the tightness, &c. across the waist, abated. Two days after the commencement of the salivation, on taking some broth, she felt a squeamishness at the stomach, and the broth was immediately thrown up; and from that day to this, Monday, March 2d, 1812, she has invariably vomited instantly whatever solid or liquid food has been taken.*

* As some opening pills remained on her stomach, it occurred to Mr. Goodwin, her surgeon, to make trial of meat made into pills: they were given, but were speedily rejected. She was not informed of what the pills were composed.

On

On January 23d, 1812, I first saw E. P. at the desire of the professional gentleman who attended her. I then learnt that she had been, at different times, attended by physicians of the first eminence, who had most judiciously prescribed; and, however flattered I felt by an appeal to my humble exertions, I nevertheless entered with much diffidence upon the treatment of a case of such long duration, and which had been under such able management. Although there was every appearance of much organic mischief, yet it seemed probable that if the extraordinary morbid acidity existing in the digestive organs could be arrested, one source of irritation would be cut off, and some relief would thus be obtained. There was clearly a very diseased secretion impairing the chylopoietic functions, and thus giving to the nerves of the stomach that great sensibility and irritability by which all food was immediately rejected. From careful inquiry, it did not appear that this idea had been put in force: it was therefore agreed, after a consultation between her surgeon and myself, that we should make trial of this mode of proceeding. The following prescription was accordingly written:

℞. Infus. Flor. Anthemid. ℥iſs.

Pulv. Rad. Ipecac. gr. j.

Potassæ Subcarbonat. ℈j. M.*

This was given as an emetic, to be worked off with cold beef tea, and repeated every second morning. With the same intention of correcting acidity and allaying irritation, pills containing gr. ij. Extracti Conii and one of the Potassa Fusa was directed to be taken every six hours, and the obstinate costiveness which prevailed was obviated by the occasional exhibition of Gamboge and Extract. Colocynth. Comp. This plan was followed till the 13th of February, when being informed that the patient imagined it gave some relief to the uneasy sensations of her stomach, the ingesta not returning so very speedily as before, a large spoonful of Aqua Calcis was ordered occasionally, and a tea-spoonful of Magnesia to be added to each quantity of beef tea taken to work off the alkaline emetic, and the Potassa Fusa was increased to gr. ij. in each of the pills taken every six hours. This antacid treatment not only suggested itself from the very great acetous secretions into the stomach, but also from a singular case of severe vomiting with great acidity cured by magnesia, as published in the 3d volume of the Med. Obs. and Inquiries, by the late Dr. Watson. Whatever relief,

* Such was the condition of the stomach, that the draught would have produced vomiting without the Ipecac., which accounts for the smallness of the quantity of this root.

however, to the sensations of her stomach E. P. might have felt, it did not appear to me that any great advantage was obtained, although she took at times from two to three grains of the Potassa Fusa; and at one time, after taking the alkaline emetic, vomiting was constantly kept up with pure beef tea and magnesia for five hours: but notwithstanding this, the last vomiting was as acid as the first.

I think it appears very clearly from this, that the great acidity in the stomach did not arise from acetous fermentation in that organ, but was actually secreted; for there was no time for the acetous fermentation to take place, considering the continued vomiting for five hours almost without intermission.* In Dr. Watson's case, it is clear there was acetous fermentation from debility of the stomach, and consequent deficiency in the powers of the gastric juice. After vomiting for a time, the mutton broth was returned in the same state in which it was taken, and the patient was subsequently cured by bark and steel. I was then convinced that the alkaline treatment would be of no essential use, seeing that the acidity was owing to a morbid action in the chylopoietic organs, resulting most probably from morbid structure. This plan, therefore, which had been submitted to with the most patient resignation, was given up in despair: the case, nevertheless, still continued to occupy a good deal of my thoughts. In turning over in my mind the whole of the symptoms again and again, more particularly the circumstances under which the vomiting commenced, which was during a course of mercury, and two days after the salivation commenced, I began to suspect that that mineral was probably in some way connected with this long-continued and very distressing symptom. I had met with no analogous case in my own practice to guide me, but on looking over my commonplace-book, I found a reference to the Med. Obs. and Inquiries for some cases, the symptoms of which were very similar to those of E. P., and which had come on under the use of mercury. I therefore read these cases with some care, and therein discovered a very strong similarity in the origin, progress, and nature, of the symptoms of the case under my care. With this conviction on my mind, I formed the resolution of urging the propriety of again resorting to a mercurial course. I took the first opportunity of stating to my patient (who resides many miles from Bedford) the desire I had that she would again make use of mercury—she readily consented.

Several years ago, Dr. Bardsley, of Manchester, published a volume of Medical Reports, in which he stated some anoma-

* See Sir John Pringle's Experiments on Alimentary Fermentation.

lous lous cases of disease arising during the use of mercury, which resisted every mode of treatment till mercury to salivation was again resorted to, when the diseases were cured. I immediately wrote to that physician, and pointed out to him the cases in the 3d and 6th vols. of the Medical Observations.* Reflecting on E. P.'s case, and recollecting the correspondence that had passed between Dr. Bardsley and myself, in reference to the cases published by Sir J. Silvester and Dr. Dobson, it occurred to me that the severity of E. P.'s present symptoms might have originated from a similar cause, viz. a deranged state of the functions of the chylopoietic organs, induced by some error during the mercurial course entered upon in 1810. After mature deliberation, the plan was immediately enforced, March 2, 1812, the mercurial ointment was ordered to be rubbed in twice a-day, and directions were given for strict confinement to bed, with other precautions, in order to prevent any injury that might possibly arise under the peculiar circumstances of the case from the use of this active mineral. In order to preserve the stomach in as much tranquillity as possible, it was agreed that nothing whatever should be given by the mouth, (except opening pills when necessary;†) and that to support the system nourishing glysters should be given. This part of the plan, however, was found to be impracticable, the glysters not remaining even with the addition of Tinct. Opii. Care too was taken that the glysters should be thrown up very quietly, to give the greater chance of their being retained by not distending the intestine too suddenly; and in the pills the simple extract of colocynth was directed, lest the aloes in the compound form might with the glyster-pipe irritate the anus too much. The mercurial ointment was used for six weeks, besides occasionally taking pills composed of Hydrarg. Submuriat. and Extract. Conii, and Hydrarg. c. creta, with Extract. Hyoscyami, when the mouth became sore.

About a week previous to the affection of the mouth, the following symptoms occurred:—She was seized about five in the morning with a most violent pain in her stomach and

---

* It is but justice to record the candid answer of Dr. Bardsley:—"I am no less struck than yourself with the coincidence between the cases you referred me to and those I have published. Such uniformity of success among different practitioners will serve to enhance the merit of the practice. I was led to adopt it from a preconceived notion, and not from authority."

† ℞. Gambogiæ, gr. iij. Rhæi, gr. xij. Potassæ Fusæ, gr. iv. Extract. Colocynth. gr. xij. Distribue ope syrupi in pilulas xij—ii vel iij pro dosi.

 bowels, bowels, which lasted three hours, when she took a cup of warm green tea, which was instantly rejected, accompanied with a good deal of glareous matter, having the appearance in some parts of it of pus. A liquid stool soon followed, which contained the same kind of matter as that which was rejected by vomiting, in the opinion of her attending surgeon. For several days previous to this she was in considerable pain across the stomach, with a great sensation of weight, which was only relieved by large doses of opium. About five or six hours after this violent paroxysm, she took two table spoonsful of mutton broth, which to her unspeakable comfort did not come up; for this was the first food of any kind that had remained upon her stomach for fifteen months. Finding this quantity stay, it was repeated every two hours for three or four days, with similar good effects, and without any other food. From that time she has been able to retain food of any light kind, such as pudding, fish, sweetbread, &c. but these still create an uneasiness at the stomach, and meat is not relished.

On the 12th of April, two days after the cessation of the vomiting, I saw my patient. Whatever might be the exact state of the disease of the chylopoietic organs, there was no doubt that its diminution had been effected by the re-introduction of mercury into the system. Impressed with this idea, and believing that although the vomiting was checked, the *causa morbi* was not entirely subdued, and as the mouth was not as yet sore, I directed some more mercurial ointment might be used every night for another week.

On the 19th of April I again saw her, when the complete effects of the mercury were visible in the constitution. The stomach continued quiet, and she was able to take a mild nourishing diet, although she had no appetite for food, but felt an inclination for it when it was presented to her. A bitter draught* was now prescribed twice a-day; and, as the bowels were extremely torpid, a suspicious circumstance, a bolus of gr. xij Extract Colocynth. Comp. was taken with each draught, and which scarcely moved the bowels once daily. From this time to May 1st, she continued nearly in the same state, except that she began to perspire most profusely, with a fluctuating state of the ptyalism, the mouth at times being free from soreness. The perspiration was so great as to make her feel faint, and to render it necessary to change her bed-linen twice in 24 hours, and the moisture dripped from them when they were wrung. She might be

* Pulv. Rad. Columb. ʒj. Rasur. Quassiæ, ℈j. Aquæ Bullient, ℥viij. Macera per horas ij et cola.

℞. Liquoris Colati, ʒx. Spt. Myristicæ, ʒj. M.

said said to be constantly in a warm bath; she was still confined to her bed, from the fear lest any check being given to the mercurial action, before that mineral was eliminated out of the constitution, might reproduce the vomiting. The bowels were still very torpid. She now took (May 1st) a draught of an infusion of quassia and senna, with the diluted sulphuric acid thrice a-day.* With the use of these medicines the perspirations gradually subsided; but they continued very profuse for a long time, and the bowels were so obstinately costive, that very large doses of very active medicines were required to keep them moderately open; till at last she took, in the course of one day, at different times of it, ʒj Pulv. Scammon., gr. vj Gambogiæ, and gr. iv Potassæ Fusæ, and had only one evacuation, and that not loose. She was now able to come down stairs, to go about the house, and take occasionally an airing in a chaise, although she felt very weak. Her stomach retains the light food she takes, but she feels an uneasy sensation after any thing of an animal kind is taken. In this state I saw her again on the 19th of June, and as she complained of feeling very weak, and as her more immediate professional attendant, who watched her with much care, assiduity, and kindness, stated the very obstinate torpor of her bowels, resisting more than the usual purgations, she was ordered an infusion of the cinchona twice a-day, with some active pills.†

May 31, 1813.—I this day saw E. P.; she still continues to retain food of the farinaceous and vegetable kind on her stomach, which she relishes, as also fruit, puddings, and sometimes bread and cheese; but after eating a meal, she feels an uneasiness about the region of the stomach; eats no meat, disliking it; is thirsty; the tongue is clean during the day, but white in the morning; sleeps generally well; has at times transient sharp pains shooting to the back from the stomach; is at times sick, with some vomiting, but rarely; feels also at

---

* ℞. Rasur. Quassiæ, ℈j. Cort. Angustur. Contus. ʒſs. Folior. Sennæ, ʒij. Aquæ Bullient. ℥viij. Macera per horas iij et cola.

℞. Liquoris Colati, ʒix. Spt. Myristicæ, ʒj. Acid. Sulph. Dil. gtt. x. M.

My late much-respected friend, Dr. Pitcairn, first suggested to me, many years ago, the utility of combining an aperient in a small dose with bitters, as they produce a better effect that way than alone. Experience has fully confirmed to me the truth of this observation.

† ℞. Pulv. Cinchonæ, ℥ſs. Aquæ Bullient. ℥viij. Stent per horas xij et cola.

℞. Liquoris Colati, ʒvj. Lactis Amygdal. ʒiij. M.

℞. Extracti Elaterii, gr. vj. Gambogiæ, gr. xxiv. Extract. Colocynth. Comp. ʒj, distribue in pilulas xxiv—ij-iij vel iv, pro dosi hora somni.

times a transient heat on the whole body ; œdematous swellings of the lower extremities,* accompanied with erisipelatous patches of inflammation, sore to the touch, have supervened; urine varies much in quantity and color; bowels still very costive; recourse is had to the pills mentioned in the margin ; † pulse about 90, weak ; her stools vary in color and consistence, and are at times very offensive; she has gained strength and flesh, and the color of the skin is perfectly healthy ; she is soon fatigued by exercise, and a hurried motion produces dyspnœa; always feels better when the stomach is empty, and the coldest liquids agree best; tea is her favorite beverage. Such is now the altered condition of her stomach, that gr. viij Ipecac. taken one day produced no vomiting, and on the following morning gr. j Antimon. Tart. added did not increase the emetic power of the Ipecac. but operated downwards.‡ Repeated the vomit a week ago, which operated a little, and brought up some fluid of a greenish color, but not acid. These vomits she took of her own accord, in consequence of feeling uneasy at the stomach. She now takes no medicine but opening pills. In May last the menses made their appearance, and returned at the interval of six weeks, but have since totally ceased. Such is the exact state of the patient at this time—a condition comparatively very comfortable to what it was previous to the re-introduction of mercury, and which allows of the exhibition of remedies for a more perfect recovery, however doubtful that may be.

---

The ink was scarcely dry from writing the communication which I have just closed to you, when your Journal for the present month came to hand, in which I find myself called upon to publish a case, in consequence, it would appear, of a paper on Ischuria, which you did me the favor to insert in your last Number; I must therefore trouble you again.

A poor woman of this town, (still alive, bed-ridden, and suffering greatly from painful disease,) had been afflicted for several years with very distressing complaints; during one period of the progress of which she declared, and this declaration is corroborated by her neighbours, that she vomited her urine. I never personally witnessed this symptom, but

---

* This symptom had occurred previous to the severity of her illness originally, but disappeared.

† ℞. Extracti Elaterii, ℥ſs. Gambogiæ, ℈ij. Extract. Colocynth. Comp. ℥iſs. distribue secundum artem in pilulas xxiv. This quantity in the dose of one or two pills is not very active.

‡ The reader will recollect, that in the first part of the case, gr. j Ipecac. produced full vomiting.

have have very frequently visited her both prior and subsequent to its occurrence. Several professional gentlemen of great respectability, with some of whom I am in the habit of very frequent communication, visited this person, and signed a declaration, which was delivered to me by one of the gentlemen, stating such a symptom to be utterly impossible. I was absent at the time of their meeting, which was a casual one, at a considerable distance from Bedford, on professional engagements. This broad and general assertion, both colloquial and in a written document, startled me, believing, as I do, that urinous vomiting has occurred in the human subject. I was thus induced to make a search into our best authors, and the result of this investigation has been a confirmation of my belief. I accordingly transmitted this result of my inquiries, instituted both from curiosity and a just respect for the opinions of my professional brethren, to your Journal, without referring to any case, under the hope, arising, I trust, from a proper professional spirit, of drawing the attention of your ingenious correspondents to the subject of urinous vomiting, that they might supply, if they thought proper, either from their experience, or reading, or both, their ideas on this symptom; *keeping in view the well-authenticated facts from the best authorities, both of ancient and modern date,* stated in the paper on Ischuria.* With respect to the patient herself, I credited the relation of those symptoms which I did not personally witness, from the extreme severity of affliction and sufferings which I and very many others had frequently witnessed; and I have as yet found no reason whatever to withdraw the credit I gave. But whatever belief may be withheld from the woman's report of her symptoms, it cannot affect the general question of urinous vomiting, which, in a physiological point of view, is principally interesting. The pledge, if it is to be considered such, for publication, was given in *private* conversation, in a similar way that a hint at publication is *publicly* thrown out in the preliminary observations to the paper in your last Number: and if it had not been so announced, however loosely, in either way, probably the case would at some period have been submitted to the public; but surely, whether pledged or not, it must be allowed to take place, to speak in the language of Alma Mater, horâ locoque consuetis.

I am, Gentlemen,

Your very obedient Servant,

*Bedford, June* 2, 1813. G. D. YEATS, M.D.

---

* Of the latter, the case published by Dr. Senter, in the Transactions of the College of Physicians of Philadelphia, is particularly deserving of attention.

